# Experiences and perceptions of conditional cash incentive provision and cessation among people with HIV for care engagement: a qualitative study

**DOI:** 10.1186/s12889-025-22266-6

**Published:** 2025-03-22

**Authors:** Julia Giordano, Jayne Lewis-Kulzer, Lina Montoya, Eliud Akama, Harriet Fridah Adhiambo, Everlyne Nyadieka, Sarah Iguna, Elizabeth A. Bukusi, Thomas Odeny, Carol S. Camlin, Harsha Thirumurthy, Maya Petersen, Elvin Geng

**Affiliations:** 1 Perelman School of Medicine, University of Pennsylvania, Philadelphia, PA USA; 2 Department of Obstetrics, Gynecology, and Reproductive Sciences, University of California San Francisco, San Francisco, USA; 3 Department of Biostatistics, University of North Carolina at Chapel Hill, Chapel Hill, USA; 4 Research Care Training Program, Center for Microbiology Research, Kenya Medical Research Institute, Kisumu, Kenya; 5 Division of Medical Ethics and Health Policy, University of Pennsylvania, Philadelphia, USA; 6 Division of Biostatistics, School of Public Health, University of California, Berkeley, Berkeley USA; 7 Division of Infectious Diseases, Department of Medicine, Washington University in St. Louis, St. Louis, MO USA

**Keywords:** HIV, Care engagement, Retention, Conditional cash transfers, Incentives, Africa

## Abstract

**Background:**

Conditional cash transfers (CCTs) have been shown to improve retention in HIV care while they are provided, but their long-term effectiveness remains uncertain and effects may be time-limited, with cessation resulting in HIV care engagement deterioration. We explored CCT experiences, perceptions, and effects after cessation to investigate potential mechanisms of this observation and better understand the psychological mechanisms behind CCTs.

**Methods:**

This qualitative study was nested within a larger trial, AdaPT-R (NCT02338739), focused on HIV care engagement in western Kenya. A subset of participants were purposively sampled from AdaPT-R participants: adults with HIV who had recently started ART, received CCTs for one year, completed one year of follow-up without missing a clinic visit, and were randomized to either continue or discontinue CCTs for one more year of follow-up. In-depth interviews were conducted by an experienced qualitative researcher using a semi-structed guide within a month of randomization. Interviews were conducted in the participants’ preferred language (Dholuo, Kiswahili, English). Data on patient characteristics, randomization dates, and clinic visit dates to determine care lapses were extracted from the AdaPT-R database. A codebook was developed deductively based on the guide and inductively refined based on initial transcripts. Transcripts were coded using Dedoose software, and thematic saturation was identified.

**Results:**

Of 38 participants, 15 (39%) continued receiving incentives, while 23 (61%) were discontinued from receiving incentives. Half were female (*N* = 19), median age was 30 years (range: 19–48), and about three-quarters were married or living with partners. Both groups expressed high intrinsic motivation to engage in care, prioritized clinic attendance regardless of CCTs,and felt the incentives expanded their decision-making options. Despite high motivation, some participants reported that cessation of the CCTs affected their ability to access care, especially those with constrained financial situations. Participants also expressed concerns that incentives might foster dependency.

**Conclusions:**

CCTs do not appear to exert their effects through motivation, but instead act through creating opportunities for better care engagement. This study helps us better understand the durability of financial incentives for HIV care engagement and support the idea that careful consideration be exercised when implementing incentives for sustainable engagement effects.

**Supplementary Information:**

The online version contains supplementary material available at 10.1186/s12889-025-22266-6.

## Introduction

Incentives in the form of conditional cash transfers (CCTs) for healthy behaviors have been widely studied, including in people living with HIV (PWH), but questions about sustainability complicate the policy pathway. Existing research shows CCTs can improve retention in care and viral suppression for PWH during the period of time they are administered. Recent work, however — including from our group in Kenya — suggests that improvements disappear when CCTs are stopped. The viability for whether or not CCTs are a feasible solution for HIV care engagement will depend in part on whether they can be adapted or supplemented with other behavioral interventions so that they can produce lasting effects.

Explaining how CCTs work for retention in HIV care help inform potential future adaptations for sustainability. One prevailing theory from psychology that is often invoked is the idea that cash incentives act as an extrinsic motivation (changes to the desired behavior are not made for its inherent value, but because of rewards offered), which operate only when they are present, but direct evidence from the HIV treatment context is missing [1]. If true, this may help motivate patients for whom lack of motivation is a barrier to care. Other explanations in the psychological literature may also be operative and should explored. For example, some evidence exists for “crowding in,” which is when an external incentive can lead to ultimate internal motivation [2]. Other mechanisms include that CCTs can convey social norms, or that CCT’s can have “recursive” effects, where engagement in care (created initially by a CCT), leads to better health and a self-identity based on being a healthy survivor of HIV, which then in turn motivates continued behaviors [3].

In this study, we use the unique setting of a randomized trial (NCT#02338739) to directly assess from participants their views on how CCTs worked. The parent study was unique in that individuals randomly received CCTs, and then some who succeeded during receipt were randomized to discontinuation. We conducted interviews before and after discontinuation to assess how individuals perceived and explained the effects of the incentives and the effects of their discontinuation, both before and after actual discontinuation. We use qualitative interviews to further explore how people perceive money to have affected day-to-day financial choices, HIV care-related anxiety, transportation costs of care, and other factors.

## Methods

### Study population and setting

This qualitative study, led by an investigator with extensive qualitative expertise (CC), was nested within a larger parent trial, AdaPT-R. Study participants in the parent trial included adults (18 years and older) with HIV who had started ART within 90 days of study enrollment. The study took place in western Kenya (in Migori and Kisumu counties) in four government health facilities and one faith-based health facility. At study enrollment, participants were randomized to one of three stage 1 lower intensity interventions: standard of care (SOC), text message reminders (SMS), or conditional cash transfers (CCTs) in the amount of approximately $4 USD for each on time (-/+ 3 days) scheduled clinic visit. If participants in any of the stage 1 arms missed a clinic visit by 14 or more days during their first year in the study, they were randomized a second time to a stage 2 higher intensity intervention. Those in the CCTs stage 1 intervention arm who did not miss a clinic visit by 14 or more days in their first year were considered successful participants who had not lapsed care. These individuals were then randomized again to either continue or discontinue CCTs and followed for one more year. A subset of participants randomized to receive CCT at baseline were purposefully selected for interviews. Participants were eligible for this sub-study if they were randomly assigned to receive CCTs at enrollment, did not lapse during their first year in the study, and were re-randomized to either stop or continue the CCT. We purposively selected participants from a subset of participants who had successfully received CCTs for a year. We used the maximum variation sampling approach, which involved choosing participants to capture a broad and diverse range of perspectives. Selection of participants from among those randomized to continue receiving CCTs and those randomized to discontinue receiving CCTs included 75% who had participated in baseline interviews and 25% who were being interviewed for the first time, while factoring in urban (Kisumu county) and peri-urban (Migori county) health facility grouping, age (18–30 and 30+) and gender.

### Data collection

Data collection took place at five health facilities in Migori and Kisumu counties from March 2017 through December 2018. The qualitative study was supervised by two study coordinators (SI and HFA) and the data was collected by a qualitative researcher (GO) whose occupation was qualitative data collection, transcription, and translation. She had over 10 years of experience in HIV-related qualitative research experience, a bachelors degree in Sociology and Anthropology, and was well-versed in active listening, putting participants at ease, and encouraging open discussion and participant confidentiality. She did not have knowledge of the participants prior to the study. The qualitative researcher was also a native speaker of the local languages (Dholuo, Kiswahili, English). The data manager provided the study coordinators and qualitative researcher with a monthly list of the eligible participants. The qualitative researcher called eligible participants over the phone, either re-introduced herself (if they had participated in a baseline interview) or introduced herself (if to be newly interviewed), provided the purpose of the call and research, and then scheduled a time for the in-depth interviews. Participants were first taken through the informed consent process and then the in-depth interviews were conducted using a semi-structured interview guide in the participants’ preferred language. The interviews were conducted in a private room at the health facility with only the qualitative researcher and participant present to ensure privacy and confidentiality. Each interview was audio-recorded and field notes were captured. Each interview took approximately 60–90 min to complete. One participant declined to participate in the interview for personal reasons. The participants were reimbursed for their time and transport. The interviews took place within three months of randomization to continue or stop the CCT, which was prior to participants’ future clinic appointments. Interview guide key topics, fully detailed in Additional File 1, included attitudes (e.g. reaction to incentive discontinuation on care engagement), experiences (e.g. influence of incentive on care attendance and engagement, challenges and barriers encountered in care engagement), and usage (e.g. how often the incentive was utilized for its intended purpose of mitigating clinic transportation costs). Interviews were transcribed and translated into English by the qualitative researcher. To ensure transcription quality, the study coordinators reviewed the first three transcripts and periodic transcripts thereafter by comparing the translation and content to the audio file. Any changes needed were discussed and made by the qualitative researcher. The audio-files were stored securely in password protected folders on password protected laptops with only limited access by the qualitative study team. Quantitative data were extracted from the parent study database, including baseline data captured at study enrollment on patient age, sex, education, marital status, distance to clinic, mode of transportation to the clinic, socio-economic factors, and if there was a lapse in care in the year following randomization to incentive continue or discontinue.

### Data analysis

Two experienced qualitative researchers (JG, JLK) coded the interview transcripts in Dedoose software (cloud-based qualitative and quantitative analysis software) with a codebook developed deductively based on the guide and refined inductively based on emerging codes identified in the initial transcripts using thematic analysis. To ensure for inter-coder reliability and consistency, the researchers cross-coded the first three transcripts and met to review and build code consensus and consistency. The two researchers then divided the remaining transcripts for individual coding, noting any difficult coding decisions. The two researchers met weekly to discuss the coding nuances to reach agreement and confirmation of appropriate coding. After coding completion, excerpts were generated and analyzed inductively to identify theme saturation, with review and input from the study coordinators (SI, HFA) and investigators (EG, MP, CC). Themes were identified pertaining to both study arms, with the exception of themes around the effects of CCTs discontinuation, which focused on the discontinued incentive arm only. Descriptive data containing participants’ sex, age, marital status, education level, walking time to clinic, mode of transport to clinic, socioeconomics, and lapses in care were extracted and summarized in Excel. Descriptive characteristics were generated to describe the populations within the CCTs continue and CCTs discontinue arms and collectively. Among those discontinued from receiving CCTs, lapse outcomes one year following randomization to stop the CCTs were compared to patient-reported perceived plans for future care in the qualitative data to examine if plans aligned with actual future care attendance in the year following their randomization. Illustrative quotes were identified for each emerging theme. Each quote excerpt includes information about patient age, sex, and study arm and whether the patient lapsed care (missed clinic visit by 14 days are more) in the year following randomization to either continue or discontinue the incentive.

### Ethics approval

The study was approved by the institutional review boards at the Kenya Medical Research Institute (KEMRI SSC No 2838) and the University of California, San Francisco (UCSF IRB No. 13-12810). All participants provided written informed consent prior to study participation. This research fully adhered to the Declaration of Helsinki.

## Results

This study included a subset of 38 participants who had received CCTs for one year from among the 517 successful non-lapsed participants. Of the 38 participants, 15 (39%) were randomized to continue CCTs and 23 (61%) were randomized to discontinue CCTs (Table 1). Half were female (*N* = 19) and median age was 30 years (range: 19–48); 29 were married or living with a partner and all 38 had at least some primary education. Taking a motorbike or walking were the most common ways sub-study participants travelled to the clinic; 32 required less than hour to walk to the clinic. Eight sub-study participants had electricity and none had running water in their homes. When exploring who lapsed in the year after randomization, 7 of 23 participants in the CCTs discontinue arm lapsed compared to 2 of 15 in the CCTs continue arm (Table 1).

**Table 1:** Participant characteristics

Characteristics	CCTs continue *N* = 15	CCTs discontinue *N* = 23	Total *N* = 38
**Sex**			
Females	7	12	19
Males	8	11	19
**Age**			
Median age	33	30	30
Range	21–47	19–48	19–48
**Marital status**			
Married/living together	14	15	29
Single/separated/divorced	1	6	7
Widowed	0	2	2
**Education level**			
Some primary	11	10	21
Some secondary	3	12	15
Some college	1	1	2
**Time to clinic in walking minutes**			
60 min or more to walk to clinic	0	6	6
60 min or less to walk to clinic	15	17	32
**Mode of transport**			
Walking	5	11	16
Bodaboda/pikipiki (motorcycle)	8	11	19
Matatu (minibus)	2	0	2
Tuk-tuk	0	1	1
**Socio-economics**			
Electricity in the home	3	6	8
Running water in house	0	0	0
Food insecure mean score (range) (0 = low & 18 = high)	6.00 (0–12)	9.57 (2–17)	8.16 (0–17)
**Lapsed care in the year following CCTs randomization to continue or stop**			
Did not lapse	11	15	26
Lapsed care	2	7	9
Missing	2	1	3

Characteristics of participants in this qualitative study who received CCTs for one year and did not lapse care and were then randomized to continue with the CCTs or discontinue the CCTs and followed for one more year. (Table 1)

Key themes from qualitative analysis are elucidated and presented below including care motivation, effects following cessation of the CCTs among the discontinue arm only, and participant recommendations on incentives.

### Internal motivation for care is strong, but CCTs also may contribute

We found high intrinsic motivation to attend clinic visits among participants, which was not changed by initiation of CCTs. Motivation to obtain and take their HIV medication, sustain good health, and survive for themselves and their families were many participants’ primary reasons for attending care.

*“It motivated me because I would spend the money for the benefit of my house; as you know*,* money is the pillar of happiness in a family.”* Male, 27, CCTs Continued, Did Not Lapse Care.

*“[The CCTs] encouraged me because at times I could even lack salt in my house so when my clinic day comes I would say to myself to go to the clinic*,* I will find money there.”* Female, 29, Migori, CCTs Discontinued, Lapsed Care.

Most participants who indicated that the CCTs would motivate, however, also indicated they would come to the clinic regardless of the CCTs. Participants who missed clinic visits in the past also expressed a strong motivation for future care attendance.

*“HIV medication is my life*,* that is why I have never missed [a visit].”* Female, 31, CCTs Discontinued, Lapsed Care.

*“I feel that nothing can stop me from coming to the clinic because I’m the one taking medication and I know how important it has been to me and so there is nothing that can prevent me from coming to the clinic.”* Female, 22, CCTs Discontinued, Lapsed Care.

*“Coming for medication was my major reason for coming to the clinic; I cannot say that I was coming because of the [CCTs]; it motivated me*,* yes*,* but it wasn’t mandatory; I have still been able to come to the clinic.”* Male, 38, CCTs Discontinued, Lapsed Care.

*“It [CCTs] motivated me to continue coming; however*,* I didn’t consider the transport alone but also my medications because my life depends on them. My number one priority is to get my medication.”* Female, 22, CCTs Discontinued, Lapsed Care.

### The prospect of stopping CCTs did not change motivation to go to clinic

Importantly, the prospect of CCT discontinuation did not change what participates said about their overall motivation to attend clinic. Broadly, participants conveyed how much they value their health above all else and would not miss a clinic appointment to obtain their HIV medication, regardless of the CCTs. When patients reported that CCTs had an encouraging effect, they often followed with stating that the underlying motivation for care remained unchanged.

*“Even if the voucher will not be available*,* I will always make sure that I don’t miss my clinic [visit]*,* this is because my life and the life of my children depend on my medication.”* Male, 40, CCTs Continued, Did Not Lapse Care.

*“[The CCTs] encouraged me*,* yes*,* but at the same time*,* my coming was not pegged on it; at time I wouldn’t get the [CCTs] but I would still come and take medication without fail.”* Female, 43, CCTs Discontinued, Did Not Lapse Care.

### CCTs acted through creating opportunities to engage in care

Participants described ways the CCTs enabled clinic attendance in the presence of other competing daily needs and therefore operated through making more opportunities possible. One highly motivated participant discussed how the CCT simplified her life by reducing need to borrow money:

*“Because I wouldn’t have suffered looking for transport to and from the clinic like I sometimes do; I can borrow transport from someone and repay them once I am from the clinic but it’s a challenge*,* it’s just that my desire for HIV medication overrides any prevailing challenge. I must work a way out to get to the clinic.”* Female, 22, CCTs Discontinued, Lapsed Care.

Many patients emphasized the general feeling that the CCTs reduced the ubiquitous challenge of, for example, transportation:

*“[The CCTs] ease my burden to some extent that would make it easier for me to come and go back home. That is the way it helped me mostly.”* Male, 23, CCTs Discontinued, Lapsed Care.

For participants who faced physical challenges reaching the clinic, the transportation incentive was especially helpful:

*“It helped me with covering up my transport cost to the hospital since I come from far and I’m not able to walk; I don’t have a proper job for now and so I always have to ensure that I have a means of getting money to reach the hospital; the [CCTs] has really been helpful.”* Male, 41, CCTs Continued, Did Not Lapse Care.

### CCTs enabled material and social engagement commitments outside of health

Aside from covering the intended purpose of transport costs, many other participants discussed how leftover money from the CCTs allowed them to purchase food and other household necessities. One participant described how taking food with his HIV medications reduced his side effects and that he used leftover money from CCTs to buy that food:

*“[The CCTs] helped so much; I could use part of the money to buy a quarter of meat and change my diet; there drugs require someone to have a good diet.”* Male, 48, CCTs Discontinued, Did Not Lapse Care.

The leftover incentive money also helped participants fulfill familial responsibilities, as described by one mother:

*I was given Ksh. 400(~$4) and two way I used Ksh. 100 (~$1) the balance of Ksh. 300 (~$3) I would use for buying sugar for my children*,* I would use it to buy food*,* silver cyprinid*,* sometimes I would buy cooking oil.”* Female, 29, CCTs Discontinued, Lapsed Care.

Other participants mentioned how the CCTs helped make up for money earned at work or by offsetting missed income.

*“It influenced my decision to come to the clinic because I couldn’t have an excuse for missing to come; I was certain of getting some money at the end of the day even if I didn’t open business that day.”* Female, 31, CCTs Discontinued, Did Not Lapse Care.

*“I felt happy because whenever I missed making profit in my business due to the appointment the voucher I received would cater for that loss.”* Female, 31, CCTs Discontinue, Lapsed Care.

### CCTs direct effects on psychological wellbeing

Many participants expressed disappointment, sadness and increased stress when the CCTs ended. Participants were saddened, troubled, and confused by having a new or re-emerging burden of finding money or a way to get to clinic appointments, including relying on other family members. The start and stop of the CCTs also sent a confusing message; participants recognized it as being there to motivate clinic attendance, yet it was then taken away. The implications of CCTs therefore have direct effects on the psychological state, wellness, and overall quality of life for participants.

*“Some part of my heart sank…no job*,* hard to find money to get to clinic; have to rely on mum and husband; but will continue with care.”* Female, 21, CCTs Discontinued, Did Not Lapse Care.

*“I didn’t feel well because I had expectations. That’s the money I had planned to use as transport to get back home.”* Male 27, CCTs Discontinued, Did Not Lapse Care.

*“You do issue us transport cash which I thought was to motivate us to come to the clinic but later you stopped it. It is generally too complicated to be understood.”* Female, 31, CCTs Discontinued, Lapsed Care.

One participant also noted a concern about perceived stigma following CCT cessation— without the incentive and the autonomy it provided, other community members may now learn about her HIV status as she turns to them to borrow money for clinic transport. That is to say, translating social capital in relationships to material support also means providing an explanation for the need to friends and family.

*“Sometimes I have to borrow money for transport to come to the clinic*,* somebody might wonder why I come to the clinic regularly and might join the dots and get to know that I am living with HIV.”* Female, 22, CCTs Discontinued, Lapsed Care.

### CCT discontinuation led to difficulties accessing care

The cessation of the CCTs led to missing clinic visits due to the inability pay for transportation to the clinic.

*“Like this past month*,* I did not visit because I did not have transport. So*,* it would help me and I would go on time and not be late. This past one I missed because I did not have enough transport.”* Female, 27, CCTs Discontinue, Did Not Lapse Care.

“*It was a challenge because I had to strategize on how I would come to the clinic; sometimes I just don’t have money to use for transport to come to the clinic; while I was still receiving the [CCTs]*,* I could sometimes borrow money from someone with the surety that I would return the money once I came back from the clinic.”* Male, 46, CCTs Discontinued, Did Not Lapse Care.

Additionally, for one participant the unexpected CCT cessation caused him to be stranded near the clinic as he looked for funds to get home:

*“I didn’t go back to Siaya on that day and so I spent three days at my friend’s house as I was looking for my means of transport back home.”* Male 27, CCTs Discontinued, Did Not Lapse Care.

Participants spoke of the ease CCTs afforded them— accessing care was no longer a problem—yet their struggle for transportation re-surfaced after the CCTs stopped, particularly among those coming from far.

*“It [CCTs] eased my burden to some extent that would make it easier for me to come and go back home. That is the way it helped me mostly… I would like for it to be reinstated because it made it easier for me to move about.”* Male, 23, Rongo, CCTs Discontinue, Lapsed Care.

### CCT recommendations by participants

When asked about incentives in the future, some participants advised against its deployment, while others wished it could be resumed. There was a feeling that if you live far and and used the funds for transport, one could easily default from their appointments, eventually become ill or die, and blame the hospital for not meeting their transportation needs if the CCTs were not resumed. Others expressed concern that the CCTs could foster dependency. Those wishing the CCTs could be resumed felt it would continue to motivate care attendance, alleviate transportation challenges, and also help with lost wages and food.

*“The truth is that people are very poor and being given transport at the facility and ease the burden that comes with living with HIV but it can create dependence.*” Female, 22, CCTs Discontinued, Lapsed Care.

*“I would like for it to be reinstated because it made it easier for me to move about.”* Male, 23, CCTs Discontinued, Lapsed Care.

## Discussion

The parent AdaPT-R study to this sub-study demonstrated that CCTs were effective at supporting care engagement while they were provided, yet, following their discontinuation, care engagement worsened. This sub-study’s findings revealed that along with high intrinsic motivation to attend clinic visits, the CCTs provided, at least for some, additional extrinsic motivation. Given high motivation, however, CCTs effects were more likely mediated by creating opportunities for care by, for example, offsetting costs of transportation, particularly among those with constrained financial situations and/or living far way. A key finding was that CCT money was used to create opportunities outside of health that enabled participants to better negotiate relationships, stigma, and support of others. These additional opportunities also entail commitments that make it difficult to redirect money to clinic once CCTs were stopped.

This study helps elucidate why patients’ care engagement may be compromised after a financial incentive is discontinued, as observed in the parent AdapPT-R study. This sub-study found that patients had high internal motivation and the best intentions to attend clinic visits, yet motivation and intent were not enough when structural barriers resurfaced after cessation of CCTs. As behavioral economics has suggested, individuals tend to make decisions based on practicalities and current circumstances, not necessarily on what is best for their well-being and future health. If resources are scarce, individuals have fewer choices and may be not be able to prioritize their health or consider the risks involved in their decisions. Unaddressed barriers to care, such as insufficient resources for transportation, may interfere with clinic attendance [4, 5]. A qualitative study in Uganda illustrated the unending challenge of finding funds to travel to the clinic, resulting in difficult life choices [6]. A study carried out in Mexico found that cash incentives were effective when in place, but, once stopped, engagement in HIV care dropped significantly [7]. The CCTs in this study were a structural intervention that expanded patients’ options and autonomy to exercise choice in decision-making while it was provided [8, 9]. The benefit of more choice options and less worry about finances was expressed by participants who felt empowered by the CCTs; it allowed them to prioritize their HIV clinic attendance with greater ease. Discontinuing the CCTs contributed to feelings of defeat when patients, who, despite internal motivation to engage in HIV care, experienced a sudden decline in their decision-making options, leaving them structurally unable to prioritize their HIV care [9].

Theorists suggest that incentivized habits can create familiarity, trust, and apparent health benefits that continue to bolster the desired behavior even after an incentive is withdrawn (“crowding-in” theory) [10]. Therefore, we anticipated that the CCTs would nudge patients toward healthy behaviors, shifting the extrinsic motivation to intrinsic motivation for care continuity [11]. However, in contrast to existing theories that focus on the effect of incentives on motivation, patients in our study reported that CCTs seem to act entirely through creating opportunities to better engage in care. CCTs enable clinic visits to allow management of logistical and economic issues, but also through management of social barriers to getting care (i.e., lessening stigma). Whether during receipt of CCT or after discontinuation, participants did not report that the CCTs augmented or diminished motivation for HIV care. While this could be due to social desirability bias, participants offered clear explanations for their motivation for care: the importance of continued health. CCTs do not appear to exert their effects through motivation, and stopping them does not appear to compromise motivation. The opportunities CCTs provide instead directly act on the economic and social conditions that determine health related (and non-health related) behaviors.

CCTs have been shown to prompt effective increases in HIV testing and linkage to care behaviors, the first steps in the care cascade [12–17]. In our study, further along the care cascade, clinic attendance continuity was difficult for patients when the structural barriers that time-limited CCTs mitigated, reemerged. In the face of persistent structural barriers, evidence suggests that CCTs may be effective when delivered consistently, but not enough to ensure sustained behavior change without their presence [18]. Opportunities created by CCT were fungible. CCTs created economic and social commitments to optimize other aspects of life outside of healthcare, such as school fees and food. This often-necessary spending was not immediately easy to reallocate back to care engagement when CCTs were discontinued, thus explaining disengagement after stopping. Interventions that address the structural barriers with long-term change will more readily support sustained clinic attendance for PWH. As the behavioral economist Thaler has advised “if you want to people to do something, make it easy” [4]. It is essential to determine what is standing in the way of a desired behavior and then clear the path [4, 5].

Giving an incentive, then taking it away, may make it more difficult for people to do what they want [5]. Incentives may “crowd out” participants internal motivation for a desired behavior and thereby undermine the long-term sustainability of that behavior [19, 20]. In the AdaPT-R parent study, the CCT was found to be effective at preventing lapses while delivered, but after removal its effects were not durable at habit formation; in fact, the study found worse engagement in HIV care than those in standard of care after the CCTs were removed [21]. Due to the high volume of lapses among those who discontinued, the AdAPT-R study speculated that people not initially helped by the CCT were unintentionally harmed by its removal [22]. The renewed hardship in accessing care may have set back further than where they were initially by fostering unsustainable economic dependency on the incentive. Pre-incentive, PWH may have been internally motivated and coped with the resources at hand. Participants then got used to the new norm of additional resources and options with the CCTs, only to have it removed without a mechanism to address their structure barriers. Without a plan or resources for transportation after its removal, participants may have been faced with making difficult choices between spending resources on life necessities or transportation to the clinic. The temporary administration of CCTs gives PWH a sense of what it’s like to be more financially secure, and then takes that autonomy away.

These results have implications regarding the sustainability and recommended usage of CCTs. CCTs may help some PWH form good clinic attendance habits while they are consistently being provided; as an intervention in addressing financial barriers to care, CCTs are effective. However, once removed, that benefit diminishes. Most participants showed high levels of internal motivation to engage in their care; even those who disengaged were motivated to re-engage in the future. Yet, the financial burdens PWH in sub-Saharan Africa face are pervasive, and without an active method of addressing these burdens (i.e. CCTs), motivation alone is not enough to remain in care. Moreover, CCTs may in fact cause additional harm if they are only given temporarily. CCTs may be most beneficial for PWH if they are given consistently to provide reliable help to address financial constraints to getting to and from the clinic. Due to the high monetary costs of giving CCTs long-term, a targeted approach that assesses which PWH would benefit the most from CCTs may be more viable. For instance, a gradual incentive phase out approach, where the CCTs are decreased as care constraint reduction is demonstrated, could be considered [23]. Other options such as home or community delivery of ART could alleviate structural issues related to care engagement without the challenge of incentive durability. One economic evaluation of home delivery of ART in Zimbabwe suggested that for ART-stable PWH, community-based models cost less for both providers and PWH and did not impact care outcomes [24]. Other studies in sub-Saharan Africa have similarly suggested that community-based ART models provide comparable care outcomes when compared to traditional facility care and may be cost-neutral or cost-effective [25–27]. It is possible that these community-delivery methods may be more cost-effective than paying for second- and third-line treatments that are necessary for patients who have missed critical appointments.

### Limitations

Only PWH who were already attending care were included in the study; thus, the PWH who may have been suffering the most from barriers to care attendance, and may have benefited most from the CCTs, were not included. In addition, participants who were receiving CCTs but were not actively engaging in care were also not able to be included in the study, and our results therefore do not reflect this perspective. Another limitation is the fact that participants were interviewed after they had received news that their CCT was to be discontinued, but before their next clinic appointment, therefore before they actually coped with not receiving the CCT. Consequently, participant responses and comments reflect their expectations and perceptions rather than their lived experiences. As with many qualitative, interview-based studies, there is always the risk of social-desirability bias influencing participants’ reports about their motivation.

## Conclusions

This study helps us better understand the durability of financial incentives in the context of HIV care engagement, particularly when incentives end. Participants in this study prioritized their health and were intrinsically motivated to remain in care even if the incentive ceased, yet once the time-limited incentives were removed, hardships to accessing care re-emerged for some participants, particularly for those with unaddressed structural barriers. CCTs do not appear to exert their effects through motivation, and stopping them does not appear to compromise motivation. Instead, CCTs appear to act through creating opportunities to better engage in care. Together with the quantitative findings in the parent AdaPT-R study, these results support the idea that careful consideration be exercised when implementing incentives for sustainable engagement effects.

## Electronic supplementary material

Below is the link to the electronic supplementary material.

Supplementary Material 1

## Data Availability

The datasets used and/or analysed during the current study are available from the corresponding author on reasonable request.

## Abbreviations

HIV: Human Immunodeficiency Virus
AdaPT-R: Adaptive Strategy for Preventing and Treating Lapses of Retention in HIV Care
CCT: Conditional Cash Transfer
ART: Antiretroviral Therapy
PWH: People with HIV
SSA: Sub-Saharan Africa
SMART: Sequential Multiple Assignment Randomized Trial
RCT: Randomized Control Trial
SOC: Standard of Care
ATE: Average Treatment Effect
CI: Confidence Interval
SMS: Short Messaging Service/ Text Message Reminders
USD: United States Dollars
SCRC: Sociocultural Research Consultants
Ksh: Kenyan Shilling
