# Supplementary material for: Experiences and perceptions of conditional cash incentive provision and cessation among people with HIV for care engagement: a qualitative study
Source: BMC Public Health. 2025 Mar 22;25:1104. doi: 10.1186/s12889-025-22266-6 (PMC11930001; doi:10.1186/s12889-025-22266-6)
Supplement: Supplementary file 1 — Supplementary Material 1 [file 12889_2025_22266_MOESM1_ESM.docx]

| Supplement 1: Selected interview guide questions and probes | |
| --- | --- |
| Experiences and attitudes related to HIV care engagement | I want to ask you about your experiences with HIV care and treatment since you were enrolled in this study about a year ago.  Initially you were told by a member of our study team that you would be offered a transport voucher for your scheduled clinic appointments, and you have not missed a scheduled clinic visit since that time.  I want to ask you about how things have been going for you since last year, with attending clinic visits. How are you feeling now, about getting HIV care and treatment? |
|  | I’d like to ask you about what you feel are the main reasons why you have been able to successfully attend all of your scheduled clinic visits. Probe: What other things do you do, or things that other people do, that help you make your appointments? |
|  | How concerned are you about your ability to make your clinic appointments in the future? Probe: Please tell me more about that. |
|  | What, if anything, do you think would make it easier for you to make your clinic appointments? |
| Perception and attitudes on barriers to care | Now I want to ask you about how other people in your community feel about getting treatment for HIV, especially your friends or family close to you. [FOR THOSE WHO COMPLETED BASELINE, SAY]: I want to ask you some of the same questions a member of our study team asked you about this last year, to see whether or how things have changed.] How do you think most of them feel about getting care and treatment for HIV? |
|  | Please tell me, what do you think are the main reasons why people do not seek care and treatment for HIV? What other barriers do people face, related to starting HIV treatment? |
|  | And what about staying enrolled in HIV care and treatment—making clinic appointments. What are the main barriers to staying in care, for most people? |
|  | What about adhering to medications—what are the main things that make it hard for people to adhere to ARV regimens? |
| Influences on care decision/clinic attendance | Let me ask you some more about that transport voucher, and about your decision whether or not to come to this clinic [name clinic] to seek HIV care and treatment.  How did it [transport voucher] affect your decision-making about whether or not to come to this clinic to get treatment for HIV? |
|  | What other issues did you consider, when you were deciding whether or not to come to the clinic for treatment? |
|  | Now I want to ask you about the time after the clinic visit you made last year, when for the first time you received the cash to reimburse you for transport costs. How you were feeling about the transport voucher at that point? |
|  | Looking back, please tell me how much influence do you think that transport voucher had, on your decision to come to the HIV clinic that day? |
|  | What were the other main reasons why you decided to come to the clinic for HIV care and treatment? Probe: Were there any other reasons? |
|  | Looking ahead to future appointments, how important is the transport voucher for your ability to make clinic appointments? Probe: What else might interfere with your ability to make appointments? |
|  | Do you feel you would be able to attend clinic even if you weren’t to receive a transport voucher? Probe: Please tell me more about that. |
| Perceptions/Influence on clinic attendance after CCT cessation |  |
|  | Thinking about the more recent clinic visit that you did not miss, how did you feel about knowing that you would not receive a transport reimbursement for that visit? |
|  | What were all the reasons why you were able to attend your scheduled clinic appointment? |
|  | Do you have any concerns about your ability to stay engaged in care in the future? Probe: Please tell me more about that. |
|  | Looking ahead to future appointments, how important would a transport voucher for your ability to make clinic appointments? Probe: What else might interfere with your ability to make appointments? |
|  | Is there anything else you’d like to share with me, about your experiences with HIV care and treatment, or about the transport vouchers offered to people in this study? |
|  | Do you feel you will be able to continue to attend scheduled clinic appointment even if you continue to not receive a transport voucher? Probe: Please tell me more about that |
